# Virtual Reality simulator for dental anesthesia training in the inferior alveolar nerve block

**DOI:** 10.1590/1678-7757-2016-0386

**Published:** 2017

**Authors:** Cléber Gimenez CORRÊA, Maria Aparecida de Andrade Moreira MACHADO, Edith RANZINI, Romero TORI, Fátima de Lourdes Santos NUNES

**Affiliations:** 1 Universidade de São Paulo, Escola de Artes, Ciências e Humanidades, Laboratório de Aplicações de Informática em Saúde (LApIS), São Paulo, Brasil.; 2 Universidade de Sao Paulo, Faculdade de Odontologia, Bauru, Brasil.; 3 Pontifícia Universidade Católica de São Paulo, São Paulo, Brasil.; 4 Universidade de São Paulo, Escola Politécnica, Laboratório de Tecnologias Interativas (Interlab), São Paulo, Brasil.

**Keywords:** Dental anesthesia, Mandibular nerve, Simulation training, User-computer interface

## Abstract

**Objectives:**

This study shows the development and validation of a dental anesthesia-training simulator, specifically for the inferior alveolar nerve block (IANB). The system developed provides the tactile sensation of inserting a real needle in a human patient, using Virtual Reality (VR) techniques and a haptic device that can provide a perceived force feedback in the needle insertion task during the anesthesia procedure.

**Material and Methods:**

To simulate a realistic anesthesia procedure, a Carpule syringe was coupled to a haptic device. The Volere method was used to elicit requirements from users in the Dentistry area; Repeated Measures Two-Way ANOVA (Analysis of Variance), Tukey *post-hoc* test and averages for the results’ analysis. A questionnaire-based subjective evaluation method was applied to collect information about the simulator, and 26 people participated in the experiments (12 beginners, 12 at intermediate level, and 2 experts). The questionnaire included profile, preferences (number of viewpoints, texture of the objects, and haptic device handler), as well as visual (appearance, scale, and position of objects) and haptic aspects (motion space, tactile sensation, and motion reproduction).

**Results:**

The visual aspect was considered appropriate and the haptic feedback must be improved, which the users can do by calibrating the virtual tissues’ resistance. The evaluation of visual aspects was influenced by the participants’ experience, according to ANOVA test (F=15.6, p=0.0002, with p<0.01). The user preferences were the simulator with two viewpoints, objects with texture based on images and the device with a syringe coupled to it.

**Conclusion:**

The simulation was considered thoroughly satisfactory for the anesthesia training, considering the needle insertion task, which includes the correct insertion point and depth, as well as the perception of tissues resistances during the insertion.

## Introduction

In light of the importance of training in the health field, including practical classes to teach medical and clinical procedures, the use of appropriate tools is crucial. Such tools can be mannequin models and other tangible objects, or computer systems that allow virtual simulations, called simulators. In addition, there are hybrid systems that combine different elements (real and virtual) to offer to students experiences that are close to those found in the clinical practice.

Simulation-based medical training tools developed via Virtual Reality (VR) techniques have increased in recent years^16^. These techniques provide three-dimensional virtual environments with real-time interaction, which include different degrees of immersion and realism, thereby allowing an accurate reproduction of tasks that are essential for health care training procedures.

VR simulating training systems, especially those involving the manipulation of medical instruments, generally use haptic interfaces. The term haptic originates from the Greek word *haptesthai*, which means “to touch”^25^. The tactile sense consists of sensations triggered when the skin is subjected to mechanical, thermal, or chemical stimuli^4^. The most common kind of haptic interface used provides force feedback, i.e, this kind allows simulating procedures that require perfect force control or pressure executed by the student.

Within the context described, this study presents the development and validation of a haptic-based VR anesthesia injection training simulator for Dentistry procedures, specifically the local anesthesia, for the inferior alveolar nerve block (IANB), the most common type of nerve block used for dental procedures.

Furthermore, IANB has a high failure rate, approximately 20-25%^13^. These failures can be classified as: anatomical (changes in nerve pathways and mandibular foramen), pathological (infection, previous surgeries, and inflammation), pharmacological (alcoholism and the use of certain substances), physiological (fear and anxiety) and also inadequate technique (improper needle insertion and beginning treatment too quickly)^13,19^.

The literature has several studies published on needle insertion simulation, such as: prostate brachytherapy^9^, cell injection^17^, types of biopsies^8,20^ and other types of anesthesia^11^. However, thus far there is only one simulator to train the administration of dental anesthesia, similar to the one developed in this study, which emphasizes visualization compared with haptic interaction^23^. The objective of this article is to present the development and validation of a dental anesthesia training simulator, specifically for the IANB. The system considers the anatomical characteristics of children aged 7 to 12 years old and the direct anesthetic technique, focusing on the inferior alveolar nerve. The simulator provides the tactile sensation of inserting a real needle in a human patient, using VR techniques and a haptic device that provides a force feedback in the needle insertion task during the anesthesia procedure.

## Material and methods

The simulator was developed considering the following steps: (1) requirements analysis for defining the computational characteristics and literature review; (2) simulator development; (3) evaluation of the simulator; (4) analysis of the results.

The Volere method^24^ was used to gather the system requirements from interviews with teachers and students from a dental education institution. A systematic review method was applied for the literature review^15^, for data collection of the state of the art regarding training tools based on VR simulation with haptic feedback.

The haptic device used (Phantom Omni^*^) captures the movements performed by the student, including rotation and position, which are then reproduced in a three-dimensional virtual environment. The device also captures the force applied to the simulator by the student and returns tactile sensations, which favor the perceived force of the virtual components involved in the training.

A subjective evaluation was applied, using questionnaires to collect information about the tool in the opinion of dentists and future dentists. The questionnaire was detailed in subsection Questionnaires and included profile, preferences (number of viewpoints, texture of the objects, and haptic device handler), as well as visual (appearance, scale, and position of objects), and haptic aspects (motion space, tactile sensation, and motion reproduction).

Regarding the visual and haptic aspects, to test statistically significant differences of the subjects’ answers to different scenarios in the tests, Repeated Measures Two-Way ANOVA (Analysis of Variance) was used twice to verify the effects of two variables or factors separately (first, Visual Aspect and Level of Experience; second, Haptic Aspect and Level of Experience), with one variable independently of the other (*Main Effects*); as well as the effect of one variable when there is dependence on one or more levels of another variable (Analysis of two variables or *Interaction Effects*). After finding the effects using Repeated Measures Two-Way ANOVA, the Tukey *post-hoc* test was selected to compare the means of pairs and to determine the causes of these effects. The average was employed to analyze the users’ preferences.

The study was approved by the Ethics Committee in Research with Human Beings, from Bauru School of Dentistry, with the number 22821113.9.0000.5417.

### Simulator development

This subsection details the development of the VR-based simulator for dental anesthesia training, for blocking the inferior right alveolar nerve and simulating the direct administration technique. This technique consists of injecting the anesthetic only in the inferior alveolar nerve^22^. The subsection was divided into the requirements gathered, three-dimensional modeling of anatomical structures, collision detection between anatomical structures and needle, and haptic human-computer interaction.

### Requirements

The requirements for the training simulator were: (1) display three-dimensional objects to realistically represent the anatomical structures (teeth, jaw, gums, tongue, facial skin) and dental instruments, such as syringes and needles; (2) accurately detect collision between the needle and the anatomical structures; (3) allow the manipulation of the physical needle or syringe and accurately reproduce those movements in the virtual environment so the student can feel the resistance when the needle traverses the anatomical structures; (4) enable the force calibration to correctly determine the resistance of the components represented; (5) add and remove objects during training, such as removing the skin to properly view the internal structures; (6) display messages to users during the training session, showing the errors (contact of the needle with the nerve, incorrect angle for insertion), the correct outcomes (correct insertion point, correct point to deposit the anesthetic solution); and (7) recording the human-computer interaction, such as the trajectories executed, structures traversed by needle, training time, which can facilitate user evaluation.

### Three-dimensional modeling

To simulate the virtual patient and the components involved in the training procedure, the following objects were modeled: facial skin, teeth, tongue, muscles, nerves, blood vessels, and bones.

The objects were devised to ensure realism of the actual structures, that is, considering attributes such as size, shape and textures of the anatomical structures, The structures must be similar to real patients (in this case, children of 7 and 12 years of age).

The colors for virtual structures are defined using illumination parameters available in the simulator, as well as size and position of the objects. The images for texture were created capturing real images (to represent bones, for example) and images from anatomy books. The skin image was detailed with pores and oiliness. These books were also used to define the shape of the structures.

Objects, such as the skin, can be added and removed using the keyboard. This functionality is available for students who desire to view the internal structures, which, in the actual procedure, is partially obtained by retracting the cheek.

### Collision detection

The collision detection adds a complexity to the simulation, because detailed three-dimensional objects (which have a large number of vertices and polygons) require increased computational processing.

Similarly, for the simulation to be close to the real procedure, the feedback of the haptic device cannot present delays. Thus, to overcome the difficulties and to provide a realistic simulation, a method called *Octree* was applied, ensuring the desired accuracy in the collision detection^14^.

This method based on *Octrees* divides the virtual environment into eight parts or octants. It then verifies in which octant the medical instrument is and measures the distance between this virtual object and each octant. The closer octant, which has virtual objects representing anatomical structures, is then divided in the next step. This procedure is repeated until it reaches a minimum distance and the collision between two objects is accurately detected considering the polygons of these objects.

### Haptic interaction

The haptic human-computer interaction, or simply haptic interaction, allows the user a two-way communication with the simulator. This interaction includes the capture of certain movements, usually of the hands, and generates haptic sensations (which allows feeling the resistance of materials when in contact with the hands or with object manipulated by hands, such as a syringe).

In the simulator, the force feedback is measured by the resistance of each anatomical structure traversed by the needle (tongue, gums, teeth, bones, muscles, and nerve). The force is calculated according to Hooke’s Law, represented by equation *Force=k(x0 – x1)*, where *k* is a constant of resistance (applied for each structure) and *x0* and *x1* are the initial (surface of the tissue) and final needle (depth inside tissue) positions in the insertion axis (usually *z* axis). Users can calibrate the resistances. The lateral forces on the needle increase when the insertion depth increases.

**Figure 1:**
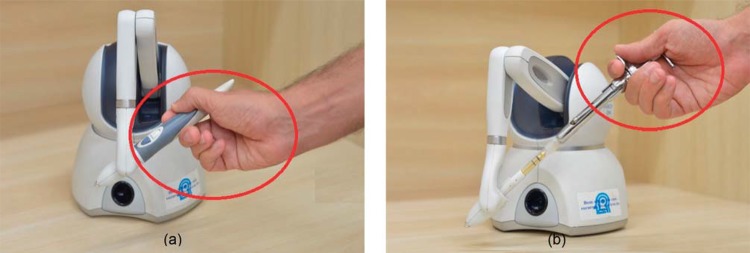
Device Handler6, 7 (a) Original stylus (b) Carpule syringe

The haptic device was adjusted by changing the handler, replacing the original stylus (Figure 1a) with the Carpule syringe (Figure 1b), the instrument that is used in the dental anesthetic procedure^6,7^.

The interaction is recorded and stored in a file and can be analyzed by an expert, for example. The information recorded comprises the paths and angles of the needle, the structures traversed by the needle and the number of times the contactoccurred. Other ways of interaction with the simulator include navigating the virtual environment and removing and adding objects that represent the anatomical structures during the procedure. The user can perform these operations using the keyboard.

### Assessing the system

A study was conducted with the participation of undergraduate students, graduate students, and teachers of a dental education institution; including students from a foreign university participating in an exchange program, totaling 26 participants. Eight different versions of the simulator were implemented to analyze the proposals, combining different possibilities to form different scenarios.

### Objective and hypotheses

The main objective of the experiments was validating the VR simulator for dental anesthesia training, the IANB procedure, especially the needle insertion task.

The following hypotheses were established:

Hypothesis 1: users prefer the virtual environment with two viewpoints, since this option enhances the user’s perception of depth of the needle insertion;

Hypothesis 2: users prefer objects with texture based on images, since they display a better realism compared with objects that only use colors for texture;

Hypothesis 3: users prefer the device adapted with a Carpule syringe, as it is the instrument used in the actual procedure;

Hypothesis 4: users report that the objects are appropriate in terms of scale, position in the virtual environment and appearance, not considering the texture (Hypothesis 2), but rather the shape of the objects.

Hypothesis 5: users report that the simulator is adequate, although the haptic device has limitations, such as the reduced workspace of the handler, small degrees of freedom of force and an insufficient force feedback for certain anatomical structures (e.g., bones).

For each hypothesis, the level of experience of the users or participants also was considered in the analysis.

### Participants

Twenty-six volunteers participated in the experiment: 2 experts, 12 at intermediate level and 12 beginners, 18 women and 8 men, all right-handed, average age of 25.3 years and an average of 5.9 years of experience in relation to the IANB procedure. In this group, three students were participants of an exchange program from a foreign university. This information is highlighted due to the possibility that students from different institutions could perform the procedure differently, but correctly, indicating that the simulator needs to predict such situations.

The users indicated their level of experience (beginner, intermediate, and expert). The participants who informed having experience in the actual procedure reported that their training basically consisted in administrating anesthesia on their colleagues and patients. One mentioned having used a cow tongue to practice.

Only one participant had experience with VR systems, including the use of a haptic device. Five participants declared having no experience in executing the anesthesia procedures and they were placed in the beginners group.

### Different scenarios

**Figure 2:**
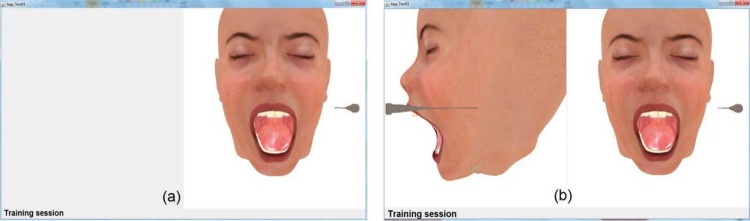
Number of viewpoints in the interface (a) One viewpoint (b) Two viewpoints

**Figure 3:**
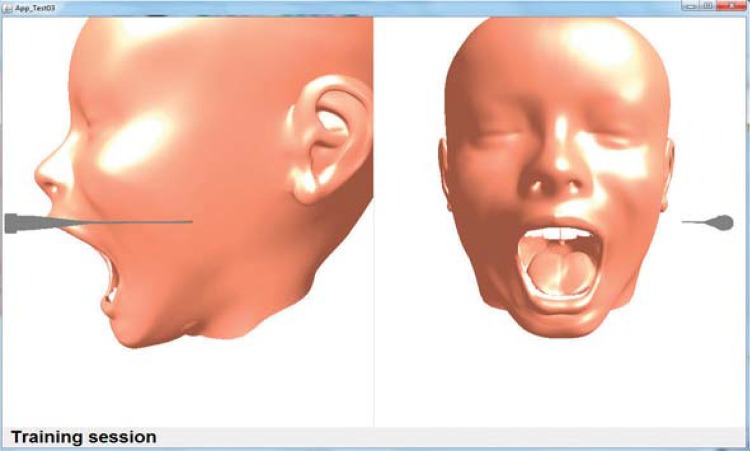
Texture of the objects with color

**Figure 4:**
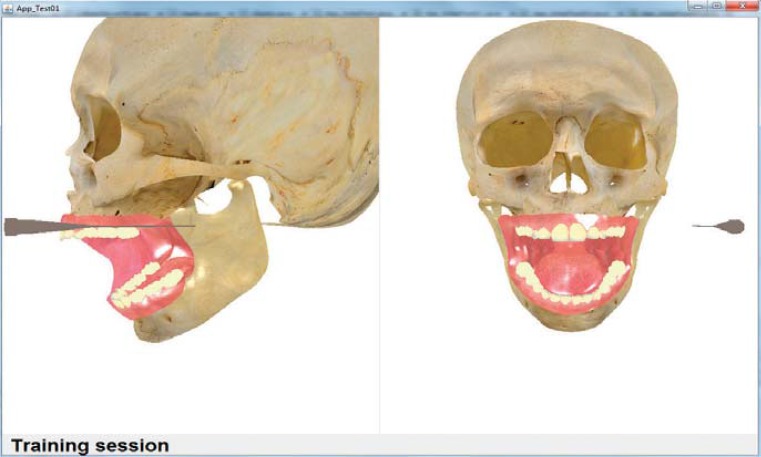
Environment with skin removal

Different scenarios were tested using combinations of the system’s versions: with one and two viewpoints (Figure 2); objects with texture based on color and image (Figures 2b and 3); haptic device, considering its original stylus and adapted version to include the Carpule syringe (Figure 1). The functionality to remove and add the object representing the skin was provided (in each version), to view (Figure 2) or hide (Figure 4) the internal structures.

### Task

The task to be performed in the virtual environment consisted of needle manipulation, inserting it at the right angle (approximately 45 degrees), in the correct spot (mucous membrane of the medial side of the branch of the mandible, towards the mandible foramen) and at the correct depth (needle tip next to the nerve) to IANB on the right side, without reaching the nerve. After performing the task in all the scenarios, the participant answered the questionnaire.

### Questionnaires

Questionnaires were designed to be applied after the tests, to collect profile, preferences, visual, and haptic aspects. They also were used to verify the participant’s sensations, such as exhaustion and understanding of the task to be performed in the virtual environment. The participants could offer suggestions, assessment, and ideas to improve the simulator.

The position of the video monitor was included in the questionnaires because the procedure is usually performed with the patient in a reclined position and the video monitor was placed in front of the participant.

The participants’ profile comprised information such as their age, gender and level of experience (beginners, intermediate, and experts) in terms of dental anesthesia procedures for IANB. In addition, the participants informed their experience with VR systems.

The preferences consisted of information on the texture of three-dimensional objects (color, image, and indifferent); the options of the haptic device handler (original stylus, Carpule syringe, and indifferent) and options regarding the number of viewpoints of the virtual environment (one viewpoint, two viewpoints, and indifferent). The term indifferent means that the other two options for each preference are irrelevant.

The realism in a simulation is important in the training situation, especially in VR systems. In these systems, the visual and haptic aspects can determine the realism^5,9,20,23^. Aspects (visual and haptic) were analyzed using the Likert-7 scale, ranging from “Strongly Disagree” to “Strongly Agree”. The visual aspects were related to three items: (1) the scale (size in the virtual environment); (2) the position, and (3) the appearance of three-dimensional objects in the environment.

The haptic aspects were related to three items: (1) the accurate reproduction of the movements (relation between virtual needle and device); (2) the quality of force feedback when anatomical structures encounters the needle, and (3) the device workspace size to perform the needle insertion movements.

## Results

**Figure 5:**
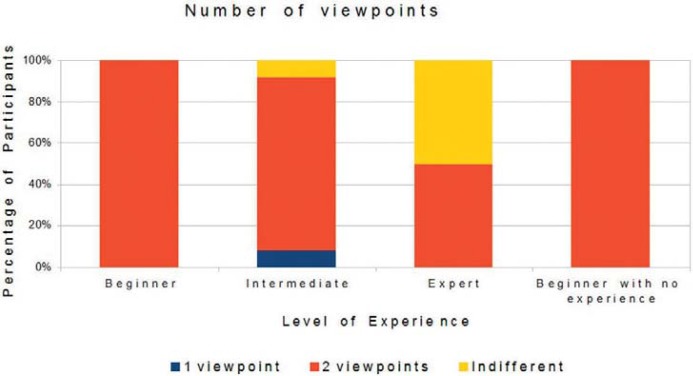
The participants’ viewpoints and experience

**Figure 6:**
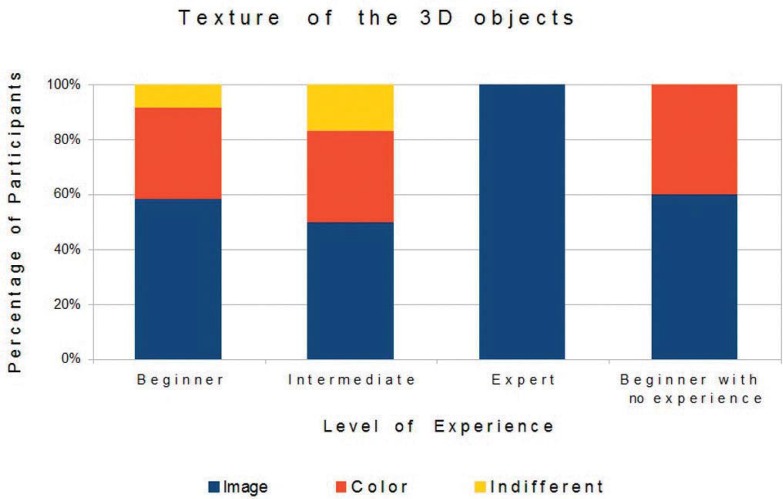
Texture of 3D objects and participants’ experience

**Figure 7:**
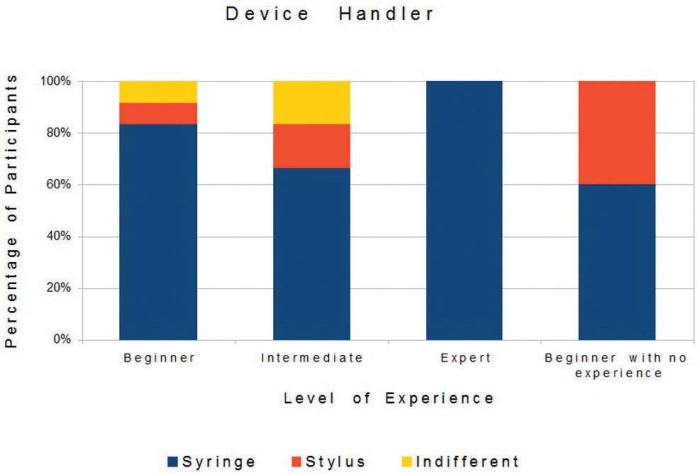
Device handler (stylus and syringe) and participants’ experience

The analysis of the participants’ preferences, obtained through the questionnaires, showed that most preferred the virtual environment with two viewpoints, objects with texture based on images and device adapted with a Carpule syringe. Figures 5, 6, and 7 show the preference-based results according to the participants’ degree of experience.

Repeated Measures Two-Way ANOVA was applied separately for each aspect (Visual and Haptic). We used the following conventional assumptions for an ANOVA test: the samples must be random and independent, the data must have a normal distribution, and the groups’ variances must be equal. Since the data of the experiments attended these conditions, this technique is considered suitable.

The analysis considered 25 participants (11 beginners, 12 at intermediate level, and 2 experts), because one participant did not answer some questions. The proposal was to evaluate the realism of the simulation based on visual and haptic aspects (three items for each aspect, according to subsection Questionnaires), as well as the level of experience. In the experiment, considering the results separately for each aspect, null hypothesis from ANOVA was false for the factor Level of Experience in the Visual Aspect (*F*=15.6, *p*=0.0002, with *p*<0.01), indicating one main effect (the experience influenced the responses). A *p-value* less than 0.01, or value less than 1%, indicates which value is significant at the 99% level. Thus, the probability of average difference between the responses of users to occur by chance is less than 1%.

According to Tukey *post-hoc* test, applied for comparing pairs of averages and showing what caused the effect, the participants of intermediate group gave higher scores in average with statistically significant differences compared with experts and beginners. In the Haptic Aspect, no statistically significant differences werefound, showing that the level of experience did not cause substantially different responses in the users’ perception in the case of the haptic interaction.

For Visual Aspect, the general average score was 5.6, considering all levels of experience, all items, and the scale (1 to 7); for Haptic Aspect, the general average score was 4.7, considering all levels of experience, all items, and the same scale. For Visual Aspect, considering all levels of experience and the same scale, the averages for each item (a, b, and c) were 5.7, 6 and 5.7, respectively. The items were (a) the scale (size in the virtual environment), (b) the position, and (c) the appearance of three-dimensional objects in the virtual environment.

For Visual Aspect, considering all levels of experience and the same scale, the averages for each item (a, b, and c) were 5.4, 4.3, and 5.2, respectively. The items were (a) the accurate reproduction of the movements (relation between virtual needle and device), (b) the quality of force feedback when anatomical structures encounter the needle, and (c) the device workspace size to perform the needle insertion movements.

The position of the video monitor did not undermine the interaction between participants and the training simulator. The anesthesia administration is usually performed with the patient reclined and the monitor was positioned in front of the student. All participants fully understood the task to be performed in the environment and feelings of fatigue during the test were minimal.

Hypotheses 1, 2, 3 and 4 were corroborated and hypothesis 5 was partially validated. The participants approved the rendering of virtual objects; however, their opinion was that the haptic feedback should be improved, changing certain force feedback values, such as the mucosa value. According to the participants, the maximum force feedback value was considered to be sufficient, especially for virtual bones.

The foreign university students performed the procedure similarly to the students of the Brazilian institution, indicating no differences regarding the anesthesia administration technique. The participants did not use the keyboard of the device to navigate, adding and removing three-dimensional objects in some situations, however, they chose to focus on the needle manipulation. They also did notice the error messages.

## Discussion

The growth in the use of VR simulators may be related to the benefits provided by them, such as: they reduce risks, preventing discomfort and complications that can be detrimental to the patients’ health^2,5^, they increase the safety of the students, who often practice on their own colleagues or patients^18,21,26^, and they allow automated performance evaluations^28^.

Moreover, these simulators use different training levels, with varying situations and difficulty levels^27^. They can minimize or eliminate infrastructure and maintenance costs of physical laboratories, which include cadavers or animals^3,10^.

The VR-based simulators have led to realistic trainings, offering advantages over the use of cadavers and animals. Although cadavers offer a physical presence, they are physiologically different from living organisms^12^. Animals have anatomical differences in relation to humans^1^. Additionally, the use of both (cadavers and animals) in training situations involves ethical issues that must be properly considered, as well as the difficulties to obtain these materials for training^3,5^.

In the virtual training context, realism is very important^5,9,20,23^. Most VR simulators have limitations to reproduce reality accurately. The haptic device used in this study has certain limitations, especially to simulate the IANB using the direct technique. The technique requires an angle of approximately 45 degrees to insert the needle and the device does not provide force feedback in six degrees of freedom (only in the x, y, and z axes). Firstly, the hypothesis was that workspace and maximum force provided by device could negatively influence the human-computer interaction because there are devices with higher workspace and maximum force than the device used.

According to the results, hypotheses 1, 2, 3 and 4 were corroborated and hypothesis 5 was partially validated. Concerning hypothesis 1 (number of viewpoints), two points are important for depth perception in a three-dimensional environment, since with one viewpoint, the participants were not able to view the needle position, especially in the z axis. For the level of experience, all beginners preferred two viewpoints.

As for hypothesis 2 (texture of three-dimensional objects), the preferred objects were those based on images, seen as more realistic, however, some participants appreciated the objects with colors. As for the texture of objects, the preferred one was based on image, and all the experts considered objects based on texture as the most suitable choice, however, beginners and intermediates gave relatively high opinion scores to color.

Regarding hypothesis 3 (the haptic device handler), manipulation via Carpule syringe was the one preferred because the syringe is the instrument routinely used by dentists to administrate anesthesia. As for the device (original or adapted), the adapted device was the preferred one, receiving high values in all the groups of levels of experience. Some beginners, with no experience, preferred the original stylus, which can be explained by the lack of experience with the Carpule syringe. It was observed that how the device is held during the needle manipulation depends on the object format (stylus or syringe), as shown in Figure 1, and holding the device handler like a pen to perform the anesthesia procedure is wrong.

Regarding hypothesis 4 (scale, position, and object format) and hypothesis 5 (space to move the device handler, motion reproduction and force feedback), which include the visual and haptic aspects, respectively, the participants preferred the virtual objects, with scale, position and formats suitable for training. They reported that the haptic feedback should be improved, with specific corrections, especially in the tactile feedback of anatomical structures, such as the mucosa. The workspace of the device, and also motion reproduction were considered satisfactory. However, other trajectories and motions will be studied, as well as other ways for improving the human-computer interaction with the haptic device used in the experiments for dental anesthesia training.

However, the VR simulator allows the user to calibrate the force feedback, specifying resistance values for each type of anatomical structure: skin, mucosa, tongue, gums, bones, muscles, and nerves. The haptic perception data of the research will be used to refine the resistance values of the structures, which will be evaluated in future tests. The maximum force feedback value provided by the device was not considered as being insufficient. Although the device does not have six degrees of freedom of force feedback, the participants did not report problems.

A question which deserves to be highlighted is that the simulator presented here considers the anatomy of children. When patients are children, the foramen is usually located slightly below the occlusal plane^19^, which is different in adult patients. Thus, the procedure in children is different due to the size of the structures in these patients. However, all the methods developed can work with objects modeled to represent any type of patient, considering any age.

This first version of the simulator can already be used for IANB training. However, the project intends to improve the system. Thus, future works include the addition of stereoscopy techniques for three-dimensional imaging with volumetric perception, other anatomical structures (adults and pathologies, for example), implementation of the anesthetic injection, and the removal of the cheek using the hand, as well as adapting the simulator to transform the system into a game, using scores during the procedure to specify success and errors.

## Conclusion

The simulator was considered thoroughly satisfactory for the anesthesia training, considering the needle insertion task, which includes the correct insertion point and depth, as well as the perception of the tissues’ resistances during the insertion. Although this research is not finished and many points will be improved in the simulator, volunteers stated that it can be used as a complementary tool in the IANB teaching procedure.

## Footnotes

*
http://geomagic.com/en/products/phantom-omni/overview
